# Editorial: Research Topic Tools, Techniques, and Strategies for Teaching in a Real-World Context With Microbiology

**DOI:** 10.3389/fmicb.2021.755500

**Published:** 2021-10-13

**Authors:** Claire L. Gordy, Melissa V. Ramirez, Micah Vandegrift, Carlos C. Goller

**Affiliations:** ^1^Department of Biological Sciences, North Carolina State University, Raleigh, NC, United States; ^2^NC State University Libraries, North Carolina State University, Raleigh, NC, United States; ^3^Biotechnology Program (BIT), North Carolina State University, Raleigh, NC, United States

**Keywords:** microbiology, bioinformatics, educational studies, open education, undergraduate learning, sharing, quantitative skills

The COVID-19 pandemic has emphasized the importance of teaching microbiology in a way that motivates learners to address global challenges and allows participants to practice skills that can be used to harness the power of the microbes around and within us. This themed issue on “Tools, Techniques, and Strategies for Teaching in a Real-World Context with Microbiology” includes twenty-five articles written by authors from around the world that describe innovative approaches for contextualizing microbiology and the impacts of these approaches on student learning (Figure 1).

**Figure 1:**
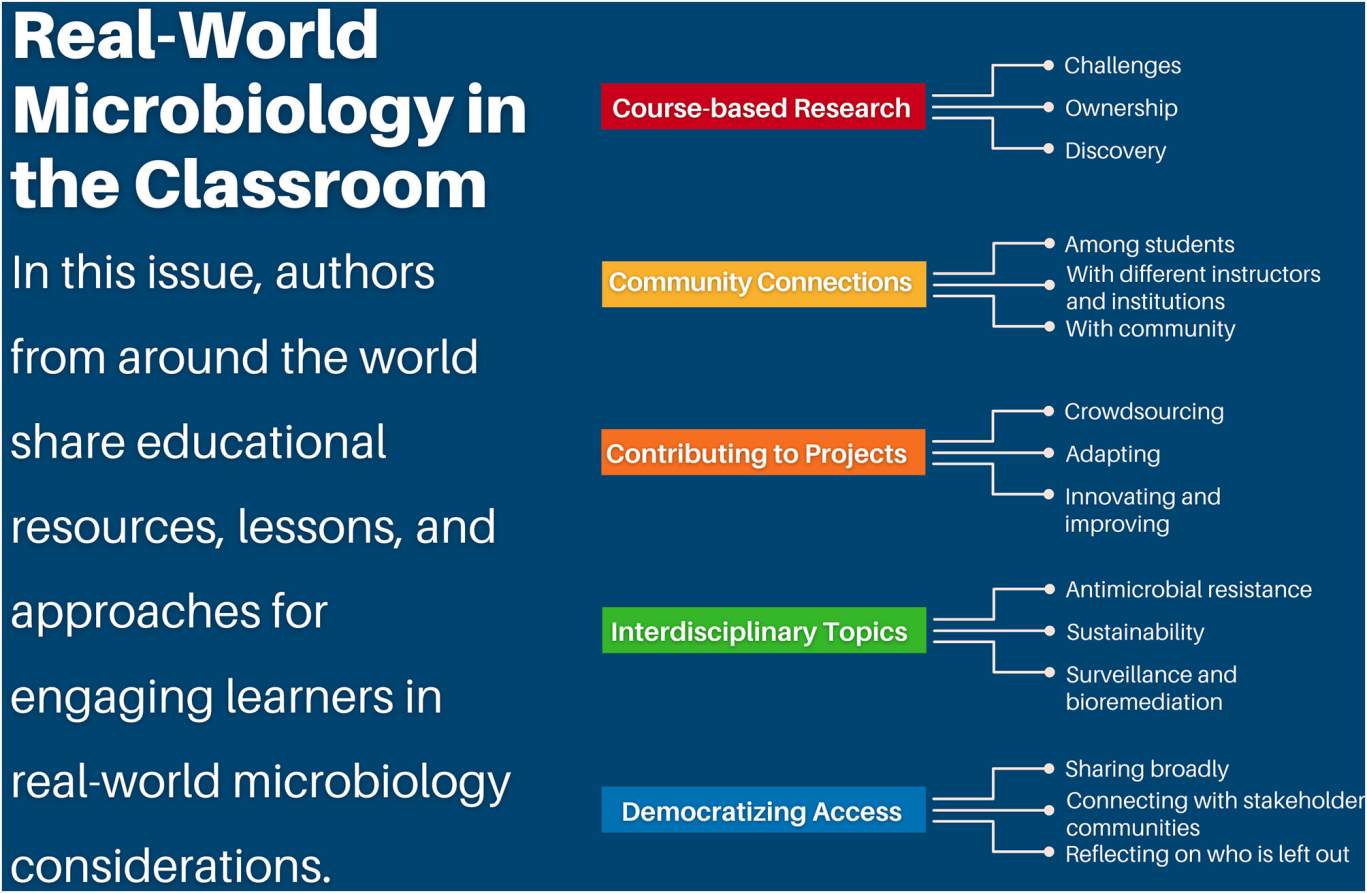
Real-world microbiology in the classroom. In this issue, authors from around the world share educational resources, lessons, and approaches for engaging learners in real-world microbiology considerations. Course-based research experiences challenge learners, foster ownership of projects, and inspire discovery. Community connections can be fostered among students, with different institutions and instructors, and with the community. Participants and educators can contribute to projects through crowdsourcing, adapting resources and projects, and innovating and improving the student experience and research methodologies. Interdisciplinary topics in this issue include antimicrobial resistance, sustainability, and surveillance and bioremediation using microorganisms. With the vision of democratizing access, educators and students can share broadly, connect with stakeholder communities, and reflect on who is left out.

Of these articles, fourteen describe lab-based courses in which learners are engaged in inquiry-based learning or Course-Based (Undergraduate) Research Experiences (CUREs/CREs) (Adkins-Jablonsky et al.; Alvarado et al.; Bueso-Bordils et al.; Furrow et al.; Gordy and Goller; Maicas et al.; Petrie; Stiemsma et al.; Sun et al.; Zelaya et al.; Fuhrmeister et al.; Fuller and Torres Rivera; Greenman et al.; Lo and Le). The learners in these courses range from first-year undergraduates to Ph.D. students, and the institutions at which they are enrolled include research-intensive (R1) universities, primarily undergraduate institutions (PUIs), and minority-serving institutions (MSIs) in multiple countries.

For example, crowdsourced and highly collaborative initiatives such as Tiny Earth offer instructors across the globe frameworks for engaging student researchers in the process of antibiotic discovery and raising awareness of the challenges of emerging antibiotic resistance. In this themed issue, Maicas et al. and Bueso-Bordils et al. describe adaptations of Tiny Earth in Spain. Alvarado et al. offer new improvements to the Tiny Earth curriculum that promise new discoveries and service-learning opportunities for participants, Aho et al. describe a CURE in which learners identified non-pathogenic *Neisseria* strains with antimicrobial activity against *Neisseria gonorrhoeae*, and Fuhrmeister et al. report a novel CURE that complements the focus of Tiny Earth on antibiotic discovery by engaging learners in environmental antimicrobial resistance surveillance. While each of these initiatives focus on the global challenge of antibiotic resistance, they each take a different approach, providing valuable insight and options for instructors interested in implementing a Tiny Earth course or incorporating aspects of antibiotic discovery and/or antimicrobial resistance surveillance into existing lab courses.

Empowering students to discover the origin of antibiotics and the prevalence of antibiotic resistance prepares new researchers in fundamental wet-lab and bioinformatics skills. Equally important, science communication training equips students to share their findings and research experiences and reflect on the process of scientific discovery. Lloyd describes how blogging activities kindle intrinsic motivation in learners in a medical microbiology course. Linares et al. report a collaborative service learning project in which students and faculty work in interdisciplinary teams to engage marginalized communities in Madrid by developing educational outreach activities using movies related to specific infectious diseases that community members indicate they would like to understand better. Sagmeister et al. describe the use of role playing activities to address antibiotic resistance and societal issues. These articles share useful experience and ways to encourage instructors to make courses more dynamic and connected to the public.

Beyond antibiotic resistance, authors report courses engaging students through the connection of microbiological concepts to multiple other topics with clear impacts on human health. Potter's students contribute to a service-learning project in which they educate their peers on handwashing and vaccination. Fuller and Torres Rivera's students interview family members to learn about traditional home remedies used within their family, and then test the efficacy of these remedies in preventing the growth of various microbes. Both of these innovative approaches allow learners to ground their scientific learning in their own lived experiences, by allowing students to connect that learning to the health habits of their campus community or to their family's traditions and culture.

Another topic with clear real-world relevance and opportunities for students to practice cutting-edge techniques is the microbiome, the genomic potential of microbial communities. The microbiome was once a complex concept that was only mentioned in a few upper-division courses. Now, however, metagenomics has been democratized through a series of educational initiatives such as the Bean Beetle Microbiome Project (Zelaya et al.). Through careful assessment of the impact of exposing students to inquiry-based learning of microbiome communities, this and other projects have highlighted the opportunities to engage students in the creation and analysis of microbiome datasets. The potential of microbiome-focused CUREs for lower-level undergraduate courses is further demonstrated by both Lo and Le and Stiemsma et al. Lo and Le report that a novel soil microbiome CURE conducted in large-enrollment introductory biology courses resulted in increased research skills as well as self-efficacy, while Stiemsma et al. report that a first-year Students as Scholars course in which learners investigated the relationship between soil salinity in coastal watershed regions and coliform contamination of water resulted in increased confidence and persistence in STEM. In addition to these descriptions of novel microbiome-focused CUREs, Muth and Caplan review educational microbiome resources that have been published. Available resources now include lessons, activities, and entire courses that can be adapted for a variety of different contexts. To increase relevance, established protocols can now be adapted to specific environments or samples that will resonate with students.

Learners in many of these microbiome-focused CUREs gain hands-on experience in environmental sampling, Next Generation Sequencing technologies, and analysis of data sets with environmental relevance. Several other articles in this issue engage learners in connecting microbiology to sustainability and environmental justice through lab-based research and classroom activities. Adkins-Jablonsky et al. report outcomes from three CUREs focused on heavy metal pollution at three different institutions. Gordy and Goller describe a CRE in which undergraduate and graduate learners collaborate to create products sustainably in yeast and explore the social, environmental, and ethical implications of metabolic engineering strategies used to produce foods, medicines, and fuels in yeast through case studies. Shay et al. share a problem-based classroom activity that brings together biochemical and microbiological concepts through which learners address the roles of microbes in degrading paper waste. The scientific concepts addressed in these three articles differ; yet learners in all of these courses gain an understanding of how microorganisms function in the environment and can be used to remediate environmental damage or create products in a sustainable manner.

Another emerging theme in the articles in this issue—both those engaging learners in microbiome research and others—is food microbiology. Petrie's students sequence natural CRISPR arrays in lactic acid bacteria in yogurt, while Gordy and Goller's students produce beta carotene in yeast and learn about the technology used to produce the Impossible Burger through a case study-based approach. Greenman et al.'s students focus on foodborne pathogens by isolating and characterizing environmental *Salmonella enterica* from stream sediments and poultry litter.

Sharing instructional materials and the results of educational studies through this issue and other publications helps build lesson plans and curricula for instructors to adapt and improve. Sharing protocols (e.g., Tiny Earth) (Bueso-Bordils et al.; Maicas et al.) and datasets (Zelaya et al.) lowers the barriers for more institutions to implement similar authentic research activities. Evaluating the impact of tools, techniques, and strategies deployed at different institutions will enrich our understanding of pedagogical choices, student engagement, and instructor needs while increasing student research output.

Beyond instructors sharing their educational materials, Sun et al. describe how they empower students to share their work in an open access online undergraduate research journal that increases access to research while providing fundamental skills to participants, such as scientific writing and peer review. In this way, students can also drive sharing and encourage others to use and build on their findings.

As we consider the rich learning experiences described in the articles in this themed issue as well as those reported in other venues, it is always important to ask, who is being left out? Are there barriers preventing the widespread adoption of these initiatives? Are marginalized populations unable to take full advantage of these resources? If so, how do we include real-world microbiology context in their educational experiences?

Several articles in this issue address some of the barriers that prevent learners and instructors at many institutions from benefiting from the rich educational experiences and activities reported throughout the issue. For example, Nguyen et al. describe a laboratory method that can be used to identify microorganisms in teaching environments that lack access to PCR, and Faist et al. describe the biocrust model system, an accessible and portable ecosystem that can be used to teach concepts ranging from microbial evolution and ecology to structure and function in a range of classroom settings and types of institutions. Smith-Keiling, along with Aparna et al., directly discuss barriers that prevent adoption of inquiry-based labs and CUREs/CREs at resource-limited institutions. Smith-Keiling addresses biosafety challenges that are common in many teaching labs, while Aparna et al. describe an approach for introducing real-world microbiology techniques in environments where learners are typically not exposed to these skills. These innovative approaches will each help to make real-world microbiology classroom experiences available to more students.

Beyond institutional resource limitations that impact instructors' ability to implement CUREs/CREs, we must begin to consider structural and systemic barriers that disparately impact the access of certain groups of students to real-world microbiology experience. The classroom—be it the lecture hall, the lab, or the online space—is not the only option for contextualizing real-world microbiology practices. Professional experiences such as internships and research opportunities for credit are transformative experiences for participants. Unfortunately, these opportunities are not available to all students due to eligibility requirements, funding, and institutional limitations. For example, many opportunities for independent undergraduate research experiences funded by US federal funding agencies exclude students who are undocumented or DACA recipients. Other students meet federal eligibility rules but not the selection criteria set by individual research experience for undergraduates (REU) programs. In particular, students who have not followed a traditional path, who may have had a rocky start to their undergraduate career but who show passion and promise, will not rise to the top of the candidate pool when criteria include GPA and other traditional metrics.

To create undergraduate research experiences that are open and accessible to all students, educators and institutions must think outside the usual framework and funding sources. Those interested in developing open and accessible research opportunities can draw on the virtual tools and connections developed out of pandemic-driven necessity to create experiences that blur institutional boundaries, allowing learners and mentors to interact and collaborate regardless of location. Open science and open educational practices can allow for more access to the guides that instructors can then use to mentor students both in person and virtually.

While creating virtual research experiences is less expensive than residential REUs, funding for student stipends is critical: unpaid research internships serve to consolidate valuable research experiences among those who can afford to work for free. Thus, PIs must seek out agencies and foundations that are able to fund student stipends regardless of factors such as immigration status. For example, Code for Science and Society's Virtual Event Fund (Virtual Event Grants, 2020) has provided funding for MORE (Mentored, Open Research Experiences), a pilot online undergraduate research experience through which students receive training in open data science practices focused on microbiome analysis and other genomic research skills, carry out mentored research projects, and receive a stipend. This approach of creating open, online resources to train learners in skills necessary for real-world microbiology research can be used not only to support cross-institutional undergraduate research programs, but also to further disseminate the innovative courses and activities published in this issue and to increase the number of learners who can benefit from them.

As you review the articles in this issue for topics of interest and initiatives you can connect with or incorporate, we encourage you to reflect on how you can expand their reach—not only by implementing them at your institution, but by thinking about how you can begin to dismantle the barriers that prevent students from enrolling in your courses, studying microbiology at your institution, or undertaking independent research. After all, sharing these educational experiences and resources benefits current students and future researchers, improving education and promoting discovery.

## Author Contributions

CCG and CLG wrote and edited the text. MR and MV conceived the open mentoring program and edited the text. All authors contributed to the article and approved the submitted version.

## Funding

CLG (PI), CCG, MR, and MV (co-PIs) received funding from Code for Science and Society in support of MORE: Mentored, Open Research Experiences.

## Conflict of Interest

The authors declare that the research was conducted in the absence of any commercial or financial relationships that could be construed as a potential conflict of interest.

## Publisher's Note

All claims expressed in this article are solely those of the authors and do not necessarily represent those of their affiliated organizations, or those of the publisher, the editors and the reviewers. Any product that may be evaluated in this article, or claim that may be made by its manufacturer, is not guaranteed or endorsed by the publisher.
